# Milk’s Role as an Epigenetic Regulator in Health and Disease

**DOI:** 10.3390/diseases5010012

**Published:** 2017-03-15

**Authors:** Bodo C. Melnik, Gerd Schmitz

**Affiliations:** 1 Department of Dermatology, Environmental Medicine and Health Theory, Faculty of Human Sciences, University of Osnabrück, Am Finkenhügel 7a, D-49076 Osnabrück, Germany; 2 Institute for Clinical Chemistry and Laboratory Medicine, University Hospital Regensburg, University of Regensburg, Franz-Josef-Strauß-Allee 11, D-93053 Regensburg, Germany; gerd.schmitz@ukr.de

**Keywords:** breastfeeding, DNA methyltransferase, epigenetic regulation, exosome, FTO, infant formula, lactation, miRNA-148a, milk, non-communicalbe diseases of civilization

## Abstract

It is the intention of this review to characterize milk’s role as an epigenetic regulator in health and disease. Based on translational research, we identify milk as a major epigenetic modulator of gene expression of the milk recipient. Milk is presented as an epigenetic “doping system” of mammalian development. Milk exosome-derived micro-ribonucleic acids (miRNAs) that target DNA methyltransferases are implicated to play the key role in the upregulation of developmental genes such as *FTO*, *INS*, and *IGF1*. In contrast to miRNA-deficient infant formula, breastfeeding via physiological miRNA transfer provides the appropriate signals for adequate epigenetic programming of the newborn infant. Whereas breastfeeding is restricted to the lactation period, continued consumption of cow’s milk results in persistent epigenetic upregulation of genes critically involved in the development of diseases of civilization such as diabesity, neurodegeneration, and cancer. We hypothesize that the same miRNAs that epigenetically increase lactation, upregulate gene expression of the milk recipient via milk-derived miRNAs. It is of critical concern that persistent consumption of pasteurized cow’s milk contaminates the human food chain with bovine miRNAs, that are identical to their human analogs. Commercial interest to enhance dairy lactation performance may further increase the epigenetic miRNA burden for the milk consumer.

## 1. Introduction

The environment in early life has an important influence on human growth and development, including the “programming” of far-reaching effects on the risk of developing non-communicable diseases in later life [1,2]. Thus, recent research focuses on the role of early nutrition for the epigenetic basis of developmental programming [3]. Milk is the first postnatal nutritional environment of all mammals from the beginning of extrauterine life to the end of the lactation period. Milk is a very complex secretory product of ancient adaptation strictly controlled by the lactation genome representing a most critical maternal regulator of offspring development [4]. Neolithic humans differ is this regard as they are persistently exposed to the milk of another species, predominantly to the milk of dairy cows.

There is accumulating evidence that milk functions as a transmitter or relay between the maternal lactation genome and epigenetic regulation of genes of the milk recipient, who under physiological conditions is the newborn infant, but under Neolithic conditions is the human consumer of bovine milk [5,6]. In fact, epigenetic processes are considered to play a pivotal role in regulating tissue-specific gene expression and hence alterations in these processes can induce long-term changes in gene expression and metabolism which persist throughout life course [7,8,9]. Because human milk protects against diseases of civilization in later life [10,11], the World Health Organization recommends exclusive breastfeeding for up to six months with continuation of breastfeeding for at least the first two years [12].

We have already suggested that milk exerts its functional role as an epigenetic imprinting system for the milk recipient [6,13]. To fulfil its function as an epigenetic regulator, milk transfers lactation-specific miRNAs, which are secreted as extracellular vesicles derived from mammary gland epithelial cells (MECs) [14]. The majority of milk-derived miRNAs are transported in exosomes, secreted nanoparticles (30–100 nm) surrounded by a stable lipid bilayer membrane protecting and ensuring long-distance miRNA transfer. Indeed, exosomes are appreciated as important factors of epigenetic regulation that modify stem cell biology [15]. Accumulating evidence supports the view that milk-derived exosomal miRNAs reach the systemic circulation of the newborn infant and the human consumer of cow’s milk (reviewed in [16,17,18,19,20,21]). To understand the role of milk exosomal miRNAs as crucial epigenetic transmitters for mother-child communication, it is mandatory to be familiar with the basics of extracellular vesicle biology.

## 2. Extracellular Vesicles: Signalosomes for Intercellular Communication

In 1946, extracellular vesicles (EVs) were first detected as procoagulant platelet-derived particles in normal plasma [22] and later on have been described as “platelet dust” [23]. In the 1980s, exosomes were described as vesicles of endosomal origin secreted from reticulocytes [24,25,26]. In the meantime, EVs have been classified into three main groups: (1) exosomes (30–100 nm in diameter) formed via the endocytic pathway; (2) microvesicles (100–2000 nm) formed by budding out of the plasma membrane in a calcium-dependent process; and (3) apoptotic bodies (>1000 nm) formed by blebbing of the plasma membrane during the process of apoptosis (recently reviewed in [27]). Exosomes are a subclass of EVs with a buoyant density of 1.10–1.19 g/mL that are enriched with tetraspanin proteins. They are assembled in intraluminal vesicles (ILVs) contained in multi-vesicular bodies (MVBs) that are released by fusing with the cell membrane. Their cargos are proteins, lipids, RNAs and miRNAs mediating intercellular communication between different cell types in the body, and thus affecting normal and pathological conditions. Their biogenesis, secretion, and intercellular interaction has recently been reviewed extensively [28]. The functional significance of exosomes lies in their capacity to transfer information to the recipient cell thereby modulating gene and cell functions [27,29]. Thus, exosomes transfer functional RNAs from a donor to an acceptor cell, analogous to hormones that can signal in paracrine and autocrine modes [30,31]. The presence of functional RNA in microvesicles was first detected in 2006 for murine stem cell-derived EVs [32]. In 2007, the uptake of mRNAs and miRNAs of murine mast cell-derived exosomes by human mast cells has been reported for the first time [33]. There is recent evidence indicating that exchange of genetic information utilizing persistent bidirectional communication mediated by stem cell EVs could regulate stemness, self-renewal, and differentiation in stem cells and their subpopulations [34,35].

## 3. Milk Exosomes: Long-Distance Transmitters of Lactation-Specific miRNAs

**Figure 1:**
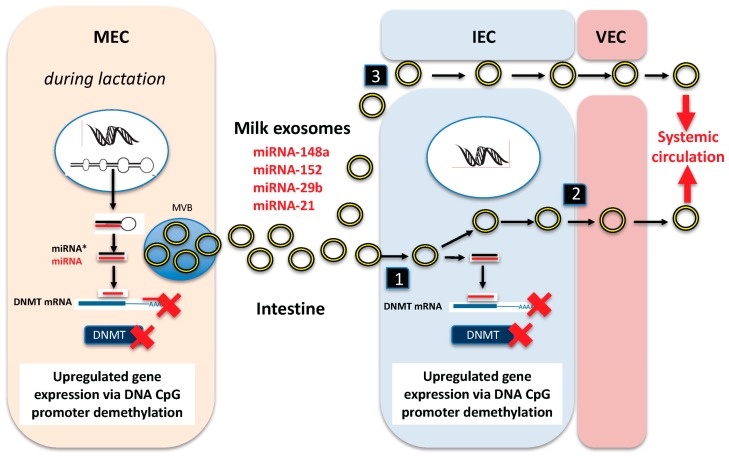
Working model of exosomal transfer of lactation-specific miRNAs that target DNA methyltransferases (DNMT) of the milk recipient. Mammary gland epithelial cells (MEC) secrete DNMT-targeting miRNAs via exosomes, which are taken up by (1) intestinal epithelial cells (IEC) and (2) vascular endothelial cells (VEC) via endocytosis; (3) Especially during the postnatal period, which is associated with high interstinal permeability, milk exosomes may travel along IEC intercellular spaces. After entry into the systemic circulation, milk exosomes may reduce DNA methylation of peripheral target cells.

Milk exosomes are regarded as indispensable signalosomes mediating cellular communication between the mother and her nursing infant [13,14,17,18,19,20,21]. In 2007, immune-regulatory exosomes from human colostrum and mature human milk have been isolated and characterized for the first time [36]. In the meantime, milk-derived exosomes have been detected in colostrum and mature milk of humans, cows, buffalos, goats, pigs, marsupial tammar wallabies and rodents [36,37,38,39,40,41,42,43,44,45,46,47,48]. Milk miRNAs have been detected in both the fat fraction of milk and skimmed milk [17,49,50]. The largest membrane-coated vesicles present in milk are the milk fat globules (MFGs) that transport the main fraction of milk lipids, predominantly triacylglycerols [51]. The major bovine milk fat globule membrane (MFGM) proteins butyrophilin, xanthin oxidase, adipophilin, and lactadherin exhibited a 15- to 30-fold reduction in abundance in bovine milk exosome membranes, demonstrating that the secretion of MFGs (lipid droplets) and milk exosomes represent two distinct secretory pathways originating from the ER directly or through the Golgi complex [42]. Nevertheless, exosome-like vesicles have recently been observed within cytoplasmic crescents of human MFGs [52]. There are at least four major cellular sources of milk exosomes: (1) direct exosome release via mammary epithelial cells (MECs) during different stages of lactation; (2) indirect exosome sequestration from MFGs; (3) exosome release by various immune-related cells in milk, and (4) exosome release via nonimmune-related cells such as milk stem cells [17,42,50,52,53,54,55,56,57]. It should be emphasized however that the majority of human milk miRNAs primarily originates from MECs resulting in unique miRNA profiles of fractionated milk (Figure 1) [14].

### 3.1. Stability of Milk Exosomal miRNAs

miRNAs are very stable and can be effectively retrieved and analyzed from formalin-fixed paraffin-embedded tissues [58,59]. Exosomal package of miRNAs serves as a special biological modification increasing their stability, an important functional feature for miRNA transfer between cells. Exosomes exhibit a rigid lipid bilayer membrane, which compared to their parent cells is enriched in cholesterol and sphingomyelin [60]. The exosome membrane serves as a protective barrier for external degradative insults. In contrast to exosome-free synthetic miRNAs, exosomal miRNAs of human milk resist harsh external conditions [43]. Acidification of bovine milk to mimic the acidic gastrointestinal tract environment did not affect RNA yield and quality [38]. miRNAs of pasteurized cow’s milk have also been shown to be stable under degradative conditions, such as RNase treatment, but were degraded by the addition of detergent, which destroys the protective lipid bilayer membrane [40,61]. In contrast to macrophage-derived exosomes, bovine milk-derived exosomes were much more stable under degrading conditions, including low pH, boiling and freezing [62]. However, pasteurization and homogenization of cow’s milk caused a substantial loss of miRNAs (63% loss of miRNA-200c, 67% loss of miRNA-29b in skim milk). In contrast, effects of cold storage and somatic cell content were quantitatively minor (<2% loss) [63]. Buffalo milk miRNA-21 and miRNA-500 were found stable under different household storage conditions indicating that these could be biologically available to milk consumers [48]. Heating in the microwave caused a 40% loss of miRNA-29b but no loss of miRNA-200c [63]. Ultra-sonication treatment of bovine colostrum exosomes abolished their immune-regulatory activity pointing to the critical role of exosome membrane integrity for exosome function [44]. Fermentation results in quantitative changes of milk-derived exosomes and reduction of milk miRNA levels [64]. Milk exosomes exhibit cross-species tolerance with no adverse immune and inflammatory responses [65]. Recently, 200–300 nm large, miRNA-223- and miRNA-125b-enriched EVs have been demonstrated in cow’s milk that also resist digestion under simulated gastrointestinal tract conditions [66].

Taken together, human and bovine milk exosomes resist harsh gastrointestinal tract conditions, important requirements for exosomal miRNA transfer into the systemic circulation (Figure 1) [19].

### 3.2. Milk Exosome Uptake

Cells are able to take up exosomes by a variety of endocytic pathways, including clathrin-dependent endocytosis, and clathrin-independent pathways such as caveolin-mediated uptake, macropinocytosis, phagocytosis, and lipid raft-mediated internalization [67,68,69,70,71]. Exosomes can easily cross endothelial barriers including the blood-brain barrier [72,73]. First uptake studies of milk exosomes have been performed with macrophages. It has been demonstrated that both human and bovine milk exosomes carrying miRNA and RNA were taken up by human macrophages [61,62,74]. Intestinal uptake of bovine milk exosomes by intestinal epithelial cells (IECs) is mediated by temperature-dependent endocytosis and depends on cell and exosome surface glycoproteins in IECs (Figure 1) [75,76]. PKH67-labeled bovine milk exosomes have been detected in IECs of the ileum and isolated splenocytes of mice that received bovine milk exosomes by daily oral gavage [75]. It has recently been demonstrated that porcine milk exosomes promoted proliferation of IECs [77]. Porcine milk exosomes significantly raised mice' villus height, crypt depth and the ratio of villus length to crypt depth of intestinal tissues [77]. In addition, human vascular endothelial cells (VECs) have also been shown to transport bovine exosomes by endocytosis [78], which supports our view that milk-derived exosomes and their miRNA cargo may reach the systemic circulation and peripheral tissues of the milk recipient (Figure 1) [6,19]. In fact, it has been reported that bovine milk miRNA-29b and miRNA-200c are dose-dependently absorbed and modify gene expression in peripheral blood mononuclear cells (PBMCs) of human milk consumers [79]. Chen et al. [77] recently demonstrated that the addition of porcine milk exosomes to IPEC-J2 intestinal cells raised intracellular levels of milk-specific miRNA-7134, miRNA-1343, miRNA-2320, miRNA-181a, miRNA-769-3p, and miRNA-128. This resulted in a significant suppression of FAS mRNA, which is a target of miRNA-2320 and miRNA-181a, and decreases of SERPINE mRNA, a target of miRNA-769-3p and miRNA-128, respectively [77].

These data suggest that milk exosome-derived miRNAs enter IECs and reach the systemic circulation [79]. There is further translational evidence supporting that milk exosomal miRNAs reach the bloodstream and modulate gene expression of the milk recipient. For instance, highly expressed lactation-specific miRNAs have been detected in the serum of the neonate wallaby (*Macropus eugenii*) in comparison to adult blood, suggesting systemic uptake of these milk-derived miRNAs [46]. Furthermore, immune-related exosomal miRNAs have been detected in higher numbers in the colostrum than in mature porcine milk [41]. The detection of higher concentrations of these immune-related miRNAs in the serum of “colostrum-only” fed piglets compared with the “mature milk-only” fed piglets further indicates the uptake of milk-derived miRNAs into the systemic circulation of the piglet [41]. Moreover, an integrated genomics and computational analysis has characterized the likelihood of milk-derived miRNAs to get transferred into the human circulation [80]. Remarkably, predicted target genes of 14 highly expressed miRNAs of bovine milk fractions were related with organismal development such as hematological, cardiovascular, skeletal, muscular, and immune system development [81] favoring a systemic gene-regulatory role of milk-derived miRNAs (functional hypothesis) [6,14,16,17,19,79,81].

It should be mentioned that the “nutritional hypothesis” of milk-derived exosomes is primarily based on three mouse models, which are inherently problematic: (1) miRNA-375 KO mice; (2) miRNA-200c/141 KO mice [82] and (3) transgenic mice presenting high levels of miRNA-30b in milk [83]. These models may all be inappropriate to study the physiological traffic of milk miRNAs to the newborn mammal as they are based on aberrant miRNA expression and did not control exosome-mediated miRNA transfer [19]. The study of Auerbauch et al. [84], which could not detect bovine milk-derived miRNAs in human plasma, may have been compromised by sample damages due to an interrupted cold chain [19].

In summary, the majority of studies supports the view that exosome-derived lactation-specific miRNAs reach the systemic circulation of the milk recipient and exert gene-regulatory functions in the newborn infant as well as the adult human consumer of cow’s milk (Table 1) [6,14,16,17,19,79,81,85].

### 3.3. Milk’s Exosomal miRNAs

In 1988, Brenner [86] hypothesized that RNAs may function as extracellular communicators involved in eukaryotic development. Secreted miRNAs represent a newly recognized most important layer of gene regulation in eukaryotes, which plays a relevant role for intercellular communication [87,88,89]. miRNAs are part of the epigenetic machinery and are predicted to regulate nearly 60% of all human genes [90,91]. miRNAs bind through partial sequence homology to the 3′-untranslated region (3′-UTR) of their target mRNAs located in the RNA silencing complex (RISC) and cause either translational block or less frequently mRNA degradation [90]. miRNAs that are enclosed by membranous vesicles, such as exosomes, play a pivotal role for horizontal miRNA transfer [89]. Milk is apparently the most efficient long-distance miRNA transmitter modifying epigenetic regulation of its recipient. It is thus not surprising that human milk contains the highest concentration of total RNAs including miRNAs in comparison to all other body fluids [92]. Exosomes are highly specialized microvesicles that transfer miRNAs to recipient cells subsequently modifying their target gene expression [30,31]. Exosomes are thus regarded as intercellular signaling organelles (signalosomes) that participate in intercellular communication [27,28,29,30,31,93,94,95,96]. Recently, it has been reported that human milk exosomes also deliver long non-coding RNAs, which have also been implicated to be involved in the epigenetic regulation of the immune system and metabolism [97].

Our insights into active sorting mechanisms of lactation-specific miRNAs into milk exosomes are still in its infancy. However, a specific repertoire of miRNAs selectively exported to exosomes indicates an active sorting mechanism [98,99]. The expression of cellular miRNAs or specific miRNA target sequences can determine the presence of miRNAs in exosomes [100]. The sumoylated protein heterogeneous nuclear ribonucleoprotein A2B1 (HNRNPA2B1) recognizes the EXOmotif (GGAG tetranucleotide) in miRNAs and thereby accomplishes their loading into exosomes [98,99]. It has also been suggested that the addition of non-templated nucleotides to the 3′end of miRNAs might promote miRNA sorting into exosomes [101]. RNA-binding protein Y-box protein 1 (YBX1) plays an important role in sorting and secretion of miRNAs into exosomes [102]. At present, two possible routes for miRNA egress via exosomes have been suggested: an Ago2-associated pathway and an RNA-binding-protein-dependent chaperone-mediated sorting pathway [102]. Notably, the chaperone-mediated pathway includes HNRNPA2B1 and YBX1 [99,102].

## 4. Epigenetic Regulation of Lactation

Lactation describes the secretion of milk from the mammary glands and the period of time that a mother lactates to feed and epigenetically program her young. During lactation MECs dramatically enhance milk protein and milk lipid synthesis. A network of genes participates in coordinating bovine milk fat synthesis and secretion. Experimental data highlight a pivotal role for a concerted action among *PPARG*, *PPARGC1A*, and *INSIG1*. Expression of stearoyl-CoA desaturase (*SCD*), the most abundant gene measured, appears to be key during milk fat synthesis [103]. Recent evidence indicates that miRNAs control the homeostatic regulation of cholesterol and triacylglycerol metabolism [104,105,106]. In comparison to nonlactating mammary glands of the Chinese swamp buffalo, the expression of miRNA-148a among other lactation-related miRNAs significantly increased during lactation [107]. In goat MECs, miRNA-148a and miRNA-17-5p have been shown to synergistically increase milk triacylglycerol synthesis via regulation of *PPARGC1A* and *PPARA*. Notably, overexpression of miRNA-148a and miRNA-17-5p promoted triacylglycerol synthesis while knockdown of miRNA-148a and miRNA-17-5p impaired triacylglycerol synthesis in goat MECs [108]. Furthermore, miRNAs are appreciated to play an increasing role in mammary gland homeostasis [109]. Recently, a miRNA-mediated crosstalk between the human placenta and the lactating breast has been suggested [110]. Upon exposure of human MECs to umbilical cord blood-derived microvesicles, microvesicle uptake was examined by fluorescence confocal microscopy associated with increased production of the milk protein β-casein [110]. Intruigingly, a recent study provided evidence that genome-wide miRNA binding site variation between extinct wild aurochs and modern cattle identifies candidate miRNA-regulated domestication genes including *MIR148a* [111].

There is recent interest in the epigenetic regulation of bovine and goat milk production for further maximization of milk yield [112,113,114]. It is of critical importance to realize that DNA demethylation of lactation-specific genes is a critical regulatory mechanism that increases gene expression for milk protein and lipid synthesis. In the lactating cow, mammary gland-specific hypomethylation of the αS1-casein gene increased casein expression [115]. Hypomethylation of casein genes during lactation have also been demonstrated in other species [113]. In accordance, abundant lactation-specific miRNAs that target DNA methyltransferases (DNMTs) are involved in the activation of lactation-related biosynthetic pathways.

miRNA-148a, miRNA-148b, and miRNA-152 are three members of the miRNA-148/152 family that share substantial homology in their seed sequence. Wang et al. [116] found that the expression of miRNA-152 significantly increased during lactation in the mammary glands of dairy cows producing high quality milk compared with miRNA-152 levels in cows producing low quality milk. The forced expression of miRNA-152 in dairy cow MECs resulted in a marked reduction of DNMT1 at both mRNA and protein levels. This in turn led to a decrease in global DNA methylation and increased the expression of two lactation-related genes, serine/threonine protein kinase *AKT* and peroxisome proliferator-activated receptor-γ (*PPARG*), whereas inhibition of miRNA-152 showed the opposite results. Furthermore, miRNA-152 enhanced the viability and multiplication capacity of dairy cow MECs [116].

Milk-related miRNAs in bovine MECs are under the influence of lactogenic hormones such as dexamethasone, insulin, and prolactin (DIP) [117]. Intriguingly, the expression of miRNA-148a was significantly elevated by DIP treatment in bovine MEC culture medium [117]. Muroya et al. [117] reported that elevated miRNA-148a levels in DIP-treated bovine MECs are associated with their increase in milk during the bovine lactation period. Importantly, DNMT1 is a direct target of miRNA-148a [118]. The expression of DNMT1 is inversely related to the expression miRNA-148a and miRNA-152 [119,120]. Furthermore, miRNA-148a directly downregulates the expression DNMT3B [121]. Furthermore, miRNA-148a directly targets the mRNAs of *ABCA1*, *LDLR* and *CPT1A*, thus attenuates cholesterol efflux, hepatic LDL uptake, and mitochondrial fatty acid β-oxidation [106].

Bian et al. [122] showed that miRNA-29s regulate the DNA methylation level by inversely targeting both DNMT3A and DNMT3B in dairy cow MECs. The inhibition of miRNA-29s caused global DNA hypermethylation and increased the methylation levels of the promoters of important lactation-related genes, including casein-α s1 (*CSN1S1*), E74-like factor 5 (*ELF5*), PPARγ (*PPARG*), sterol regulatory element binding protein-1 (*SREBP1*), and glucose transporter-1 (*GLUT1*). The inhibition of miRNA-29s reduced the secretion of lactoprotein, triacylglycerols and lactose by dairy cow MECs. The treatment of dairy cow MECs with 5-aza-2′-deoxycytidine decreased the methylation levels of the *MIR29B* promoter and increased the expression of miRNA-29b [122]. The enhancement of lactation activity and milk yield was thus associated with upregulation of DNMT3A- and DNMT3B-targeting miRNA-29s. In fact, the miRNA-29 family (miRNA29a, miRNA29b, and miRNA29c) has intriguing complementarities to the 3′-UTRs of DNMT3A and DNMT3B, two key *de novo* methyltransferases involved in DNA methylation [123]. The Ets transcription factor ELF5 is essential for normal alveolar development and lactation [124]. Notably, an increase in ELF5 expression was associated with decreasing *ELF5* promoter methylation in differentiating HC11 mammary epithelial cells [125]. Similarly, purified MECs from mice had increased ELF5 expression and decreased promoter methylation during pregnancy [125].

miRNA-145 is another miRNA epigenetically involved in the regulation of metabolism of fatty acids in goat MECs [126]. Overexpression of miRNA-145 increased the transcription of genes associated with milk fat synthesis resulting in the expansion of the lipid droplet compartment, increase of triacylglycerol accumulation, and upregulation of the proportion of unsaturated fatty acids. In contrast, silencing of miRNA-145 impaired fatty acid synthesis [126]. Remarkably, inhibition of miRNA-145 increased methylation levels of *FAS*, *SCD1*, *PPARG*, and *SREBP1*. Notably, insulin-induced gene 1 (*INSIG1*) is a direct target of miR-145. INSIG1 binds the sterol-sensing domain of SREBP cleavage-activating protein (SCAP) and facilitates retention of the SREBP/SCAP complex in the endoplasmic reticulum [127]. miRNA-145-mediated downregulation of INSIG1 thus promotes cholesterol biosynthesis.

miRNA-21 is another abundant miRNA of cow’s milk, which indirectly inhibits DNMT1 expression by targeting Ras guanyl nucleotide-releasing protein-1 (RASGRP1) [118]. Furthermore, miRNA-21 targets multiple tumor suppressor genes (PTEN, Sprouty) and inhibitors of translation (PDCD4) resulting in the upregulation of mTORC1-mediated translation [6,128,129].

Whereas lactation-specific miRNAs directly target critical genes involved in the gene-regulatory network of lactation including milk protein and milk lipid synthesis, it is of utmost importance to realize that these lactation-associated miRNAs via targeting DNMTs demethylate critical DNA promoter regions of lactation-related genes including *CSN1S1*, *ELF5*, *PPARG*, *SREBP1*, and *GLUT1*. The generation of DNMT-targeting miRNAs (miRNA-152, miRNA-148a, miRNA-29, miRNA-21) is thus a fundamental epigenetic mechanism increasing lactation-specific gene transcription thereby enhancing lactation performance as well as milk yield in domestic animals. A genetic selection of domestic cows with increased activity of DNMT-targeting miRNAs may thus enhance milk protein and lipid yield [111].

## 5. DNMT-Targeting miRNAs of Milk: Activators of the Recipient’s Epigenome

miRNA-148a-3p is by far the most abundant miRNA detected in human milk, bovine colostrum and bovine mature milk, porcine colostrum and porcine mature milk [37,40,41,43,130]. Notably, miRNA-148a is highly expressed in human and bovine milk fat [49,130] and has been measured in substantial amounts in bovine skim milk and human milk exosomes [43]. It is possible that MFGs of nonpasteurized cow’s milk release miRNA-148a carried in crescent exosomes [52]. It has been reported that miRNA-148a-3p levels increased after homogenization and thus pressure-induced dispersion of MFGs [130].

The miRNA-29 family (miRNA-29a, miRNA-29b and miRNA-29c) has been detected in bovine colostrum and bovine milk [40,44,64,79]. miRNA-29b levels in PBMCs of healthy volunteers increased in a dose-dependent manner after consumption of pasteurized cow’s milk [79].

miRNA-21 is a major miRNA of human milk exosomes [43], human milk fat [49], human, bovine, and buffalo milk as well as porcine colostrum and porcine mature milk [37,41,65,131]. It is of critical importance to appreciate that the nucleotide seeding sequences of miRNA-148a-3p, miRNA-21-5p, and miRNA-29b-1-3p of *Homo sapiens* and *Bos taurus* are identical (mirbase.org) (Table 2). This allows the suppression of human DNMTs by these abundant miRNAs of human and bovine milk.

There are two interactive mechanisms of DNA demethylation: (1) *passive demethylation* through inhibition of DNA methyltransferases (DNMTs) and (2) *active demethylation* mediated by ten-eleven-translocation (TET) 2 and 3 [132]. TET2 binding to CpG-rich regions requires the interaction of TET2 with the protein IDAX (inhibitor of DVL/axin complex also known as CXXC4) [133]. Intriguingly, the CXXC DNA-binding domains can bind unmethylated DNA and recruit TET2 via IDAX [134]. Thus, milk-derived miRNA-mediated DNMT inhibition may promote further active TET2-mediated DNA demethylation, a critical epigenetic mechanism promoting milk-controlled gene expression.

## 6. Activation of Developmental Genes via DNA CpG Demethylation

### 6.1. FTO

Milk, the postnatal nutritional environment of the growing infant, should provide sustained mechanisms activating both transcription and translation. Circumstantial evidence supports milk’s role as an activator of *fat mass- and obesity-associated protein* (FTO)-driven transcription as well as *mechanistic target of rapamycin complex 1* (mTORC1)-mediated translation [135,136]. FTO is a *N*^6^-methyladenosine (m^6^A) demethylase that selectively removes m^6^A modifications from target mRNAs [137,138,139,140]. The m^6^A modification in mRNA is extremely widespread, and functionally important because it modulates the eukaryotic transcriptome via influencing mRNA splicing, export, localization, translation, and stability [139]. RNA m^6^A modifications are involved in the regulation of diverse RNA functions including development, cell reprogramming and circadian rhythm [140,141]. In general, the m^6^A mark of RNA is regarded as a negative regulator of gene expression and protein translation [142,143,144]. Notably, m^6^A peaks at the stop codons and 3′-UTRs, which are recognized by the human YTH domain family 2 (YTHDF2) to regulate mRNA degradation [143]. Furthermore, m^6^A blocks mRNA binding to the mRNA stabilizer human antigen R (HuR) [143].

**Figure 2:**
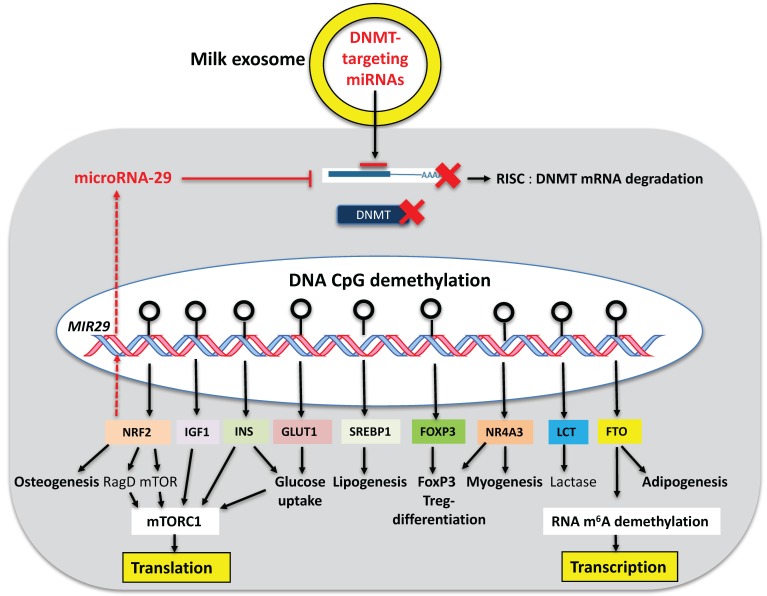
Working model of milk-mediated epigenetic regulation. Milk exosome-derived DNMT-targeting miRNAs enhance DNA promoter demethylation of critical CpG islets involved in the upregulation of gene expression of pivotal transcription factors (NRF2, SREBP1, FOXP3, NR4A3) and key metabolic regulators (INS, IGF1, CAV1, GLUT1, LCT) and the RNA m^6^A demethylase FTO. Milk-derived DNMT-targeting miRNAs may thus play a fundamental role in epigenetic enhancement of transcription and translation (see list of abbreviations).

Notably, loss-of function mutation of the m^6^A demethylase FTO is associated with postnatal growth retardation in humans [145]. In accordance, *FTO* knockdown in mice decreased body weight, altered metabolism, and retarded growth [146], whereas FTO overexpression in mice led to a dose-dependent increase in body and fat mass, increased food intake resulting in obesity [147]. There is compelling evidence that FTO plays a pivotal regulatory role for postnatal growth and energy expenditure [146,147,148,149,150]. Overexpression of FTO resulted in global decrease of m^6^A in RNAs [137]. It is known that single nucleotide polymorphisms (SNPs) in the first intron of *FTO* are associated with increased body weight, adiposity and type 2 diabetes mellitus (T2DM) [151,152,153]. Recent evidence indicates that not only *FTO* SNPs result in increased FTO activity but also epigenetic modulations associated with demethylation of specific CpG sites in the first intron of *FTO* [154,155,156,157]. Milk-derived DNMT-targeting exosomal miRNAs (miRNA-148a, miRNA-152, miRNA-21, miRNA-29s) may play a pivotal epigenetic role in reducing CpG methylation of critical gene regulatory sites of *FTO* resulting in increased FTO expression required for increased postnatal mRNA transcription (Figure 2) [135].

### 6.2. NRF2

mTORC1, the nutrient-sensitive kinase, which increases milk-induced translation [136] and anabolism [158] is closely associated with FTO activity [159,160]. FTO couples the availability of leucine, a critical activator of mTORC1 [161], to leucyl-tRNA synthase-mediated activation of mTORC1 [159,160]. Notably, the link between amino acid availability and mTORC1 signaling is dependent upon the demethylase activity of FTO [159]. Milk-mediated epigenetic activation of *FTO* gene expression may thus augment downstream mTORC1 signaling.

There are further potential milk-mediated epigenetic mechanisms that activate mTORC1 and mTORC1-driven translation. Nuclear factor erythroid 2-related factor 2 (NRF2) is an important transcription factor, which is under epigenetic control. DNMT inhibition increased NRF2 at both messenger RNA and protein levels via *NRF2* DNA demethylation [162]. NRF2 is a direct transcriptional activator of the *MTOR* gene [163], which codes the core component of the mTORC1 and mTORC2 complexes [164]. NRF2 also upregulates RagD, a small G-protein activator of mTORC1 [165,166]. Moreover, NRF2 activates miRNA-29-coding genes [167,168], which further attenuate DNMT3B expression causing further feed forward epigenetic upregulation of NRF2 expression (Figure 2).

### 6.3. INS

Insulin is a pivotal activator of anabolic PI3K-mTORC1 signaling [169]. Milk consumption results in increased postprandial plasma levels of insulin [136]. Remarkably, insulin gene (*INS*) expression is regulated by DNA methylation [170]. Kuroda et al. [170] demonstrated that β-cell-specific demethylation of *INS* enhances insulin expression, whereas DNA methylation suppresses insulin expression. Notably, the *INS* promoter is specifically demethylated in insulin-producing cells. *INS* promoter CpG demethylation may play a crucial role in β-cell maturation and tissue-specific insulin gene expression. Milk exosomal DNMT-targeting miRNAs (miRNA-148a, miRNA-21 and miRNA-29s) may thus enhance insulin secretion required for mTORC1-driven translation and anabolism (Figure 2). In fact, murine miRNA-29a was recently reported to be a positive regulator of insulin secretion in vivo [171].

### 6.4. IGF1

Insulin-like growth factor-1, the sister hormone of insulin, is the strongest growth factor and promotes mTORC1 signaling [169]. Milk consumption significantly increases serum levels of IGF-1 [172] and is associated with increased linear growth of children consuming cow’s milk [173]. Since IGF-1 controls postnatal growth, Ouni et al. [174] tested whether the CpG methylation of the two promoters (P1 and P2) of the *IGF1* gene is a potential epigenetic contributor to the individual variation in circulating IGF-1 and stature in growing children. The methylation of a cluster of six CpGs located within the proximal part of the *IGF1* P2 promoter showed a strong negative association with serum IGF-1 and growth [174]. Methylation of CpGs in the P2 promoter is negatively associated with the increased transcriptional activity of P2 promoter in patients' PBMCs following GH administration [175].

Taken together, accumulating translational evidence underlines the physiological role of milk miRNA-mediated epigenetic upregulation of critical activators of mTORC1, such as mTOR, FTO, insulin and IGF-1, which all increase in a DNA demethylation-dependent manner (Figure 2).

### 6.5. CAV1

Caveolin 1 (Cav-1) is a plasma membrane microdomain-associated protein with the capacity to modulate signaling activities in a context-dependent fashion. Cav-1 interacts with insulin receptor (IR) and IGF-1 receptor (IGF-1R) stimulating IGF-1- and IR signal transduction. Cav-1 binds to low-density lipoprotein (LDL) receptor-related protein 6 (LRP6) to generate an integrated signaling module that leads to the activation of IGF-1R/IR that finally enhances Akt–mTORC1 signaling [176,177,178]. Remarkably, demethylation of exon 1 and first intron of the Cav-1 gene (*CAV1*) is accompanied by a strong induction of Cav-1 expression [179], which has been observed during adipocyte differentiation [179]. It is conceivable, that milk miRNAs that target DNMTs may enhance insulin-, IGF-1-, and mTORC1 signaling via epigenetic stimulation of Cav-1 expression, which may enhance milk exosome uptake.

### 6.6. FOXP3

FoxP3, the master transcription factor of regulatory T cells (Tregs), plays a key role in Treg function, which is strongly related to the induction of tolerance against self-antigens (prevention of autoimmunity) and environmental allergens including food allergens (prevention of allergy) [180,181,182]. Adymre et al. [36] presented first evidence that the addition of isolated human milk exosomes to PBMCs increased the number of FoxP3+ Tregs. FoxP3 acts as a lineage-specifying factor that determines the unique properties of these immunosuppressive cells [183]. Epigenetic modifications in the CpG-rich *Treg-specific demethylated region* (TSDR) in the *FOXP3* locus are associated with stable FoxP3 expression [184,185]. Notably, stable FoxP3 expression was found only for cells displaying enhanced TSDR demethylation [183]. Recently, a linear correlation between FoxP3 expression and the degree of TSDR demethylation has been confirmed [186]. Thus, TSDR is an important epigenetic site regulating FoxP3 expression. Epigenetic imprinting in this region is thus critical for the establishment of a stable Treg lineage [183,184,185,186,187]. In contrast, hypermethylation of *FOXP3* has been associated with reduced Treg function and development of allergy [188,189]. Notably, atopic individuals express lower numbers of demethylated FoxP3+ Tregs [190].

DNMT1 and DNMT3b are associated with the *FOXP3* locus in CD4+ T cells [191]. TSDR demethylation using the DNMT inhibitor 5-aza-2′-deoxycytidine resulted in strong and stable induction of FoxP3, indicating that epigenetic regulation of *FOXP3* can be predictably controlled via DNMT inhibition to generate functionally stable Tregs [192]. Milk-derived exosomal miRNAs that target DNMT1 (miRNA-148a, miRNA-21) and DNMT3B (miRNA-148a, miRNA-29b) have been suggested to play a fundamental epigenetic role for milk-induced FoxP3 expression and Treg stabilization [130,193,194]. Thus, there is accumulating evidence that milk exosomal miRNAs are pivotal epigenetic regulators that shape intestinal and systemic immunity [36,193,194,195].

A recent epigenetic pilot study in Dutch children showed general DNA hypermethylation in the group of children with cow’s milk allergy (CMA) compared to healthy control children [196]. *FOXP3* TSDR demethylation was significantly lower in children with active IgE-mediated CMA than in either children who outgrew CMA or in healthy children [186]. Maternal rat milk in comparison with miRNA-deficient artificial formula increased mesenteric lymph node FoxP3 expression and decreased serum IgE levels to β-lactoglobulin in allergy-prone rat pups [197]. Thus, milk miRNAs operate like DNMT inhibitors that increase the stability of *FOXP3* gene expression enhancing the suppressive capacity of Tregs, promoting an immune tolerance environment [198]. This perception is in accordance with our vision that milk exosomal miRNAs shape the epigenetic environment of FoxP3-driven tolerance induction, a key mechanism for the prevention of autoimmunity and allergy [193,194,199]. It is conceivable that the availbility of the Treg-inducer milk provides a tolerogenic environment that is important for the tolerance of foreign food antigens encountered with the introduction of solid food in infants.

### 6.7. NRA4

The NR4A subfamily includes orphan nuclear receptors that belong to the larger nuclear receptors (NRs) superfamily of eukaryotic transcription factors that act as molecular switches in gene regulation modulating a complex network of cellular signaling pathways [200]. Importantly, NR4As including NR4A1, NR4A2, and NR4A3 are reported to regulate Treg cell development through activation of *FOXP3* [201,202]. NRA4 receptors directly activate the promoter of *FOXP3* and forced activation of NRA4 receptors promoted the Treg developmental program [202,203,204]. NRA4 transcription factors are thus regarded as nursing factors for the development of Tregs [205].

Milk, the epigenetic nursing system for immune cell and Treg development, may be able to promote NR4A expression. For instance, promoter CpG demethylation combined with histone hyperacetylation elucidated a regulatory mechanism that increased luteinizing hormone receptor (*LHR*) gene expression [206]. Intriguingly, NR4A3 was epigenetically silenced by NR4A3 promoter methylation, whereas NR4A3 promoter demethylation increased its expression [207]. Furthermore, NR4A3 transcription is induced by inhibition of the histone deacetylases HDAC1 and HDAC3 [208]. DNMT1 is known to associate and interact with HDACs [209,210]. The recruitment of *methyl CpG binding protein 2* (MeCP2) to methylated CpG dinucleotides represents a major mechanism by which DNA methylation can repress transcription. MeCP2 silences gene expression partly by recruiting HDAC activity [211]. For instance, transcriptional silencing from the H19 imprinting control region involves recruitment of MeCP2 and HDAC activity [212]. Milk miRNA-148a-mediated suppression of DNMT1 may thus impair the binding of MeCP2 and thus HDAC recruitment resulting in histone hyperacetylation thereby promoting the expression of developmental genes such as the NR4A subfamily of orphan nuclear receptors (Figure 2).

### 6.8. NFKBI

Milk has important effects on neonatal intestinal health and reduces the risk of necrotizing enterocolitis (NEC) in preterm infants [213]. NEC is associated with increased serum levels of proinflammatory cytokine resulting from activation of the nuclear factor kappa B (NF-κB) pathway such as interleukin-1 (IL-1) and tumor necrosis factor-α (TNFα) [214]. In an NEC model using Caco-2 intestinal cells, human milk supernatant inhibited the expression of proinflammatory cytokines IL-1β, IL-6 and TNFα [215], which are upregulated by activated NF-κB signaling. Nuclear factor κ of light polypeptide gene enhancer in B-cells inhibitor-α (IκBα) is a critical inhibitor of NF-κB. IκBα inhibits NF-κB by masking the nuclear localization signals of NF-κB proteins and keeping them sequestered in an inactive state in the cytoplasm [216]. Furthermore, IκBα blocks the ability of NF-κB transcription factors to bind to DNA, which is required for NF-κB’s proper functioning [217]. Notably, IκBα expression is higher in IECs lacking DNMTs than in IECs with active DNMT expression [218]. The CpG methylation status in the promoter of *NFKBI* exerts strong influences on NF-κB signaling of IECs [218]. Hypomethylation of the promoter of *NFKBI* increases IκBα expression, whereas methylation down-regulates IκBα expression. Absence of DNMTs in IECs resulted in lower NF-κB activation [218]. It is thus conceivable that milk miRNA-mediated down-regulation of DNMT expression exerts anti-inflammatory epigenetic modifications that enhance IκBα-mediated suppression of pro-inflammatory NF-κB signaling. In this regard, milk-miRNA-mediated upregulation of IκBα resembles the anti-inflammatory action of glucocorticoids, whose mode of action is the promotion of IκBα expression [219,220].

### 6.9. LCT

In the great majority of mammals, the intestinal expression of lactase fades after weaning except for mutant humans exhibiting *lactase persistence*. It is not known how the lactase gene (*LCT*) is dramatically downregulated with age in most individuals but remains active in others. According to recent evidence epigenetically controlled regulatory elements account for the differences in lactase mRNA levels among individuals, intestinal cell types and species [221]. There is good evidence that the persistence of high intestinal lactase activity into adult life is attributable to transcriptional regulation of *LCT* [222]. In Europeans, a single nucleotide substitution (-13910C>T) with intron 13 of *MCM6*, the gene adjacent to *LCT*, is strongly associated with lactase persistence. This substitution resides within an enhancer of *LCT* increasing promoter activity [223,224,225,226]. Seven epigenetically regulated regions have been identified that include the *LCT* enhancer located within intron 13 of *MCM6*, which house the functional DNA variants [221]. Low *LCT* transcription observed with the *lactase nonpersistence* haplotype was associated with a higher methylation status of intron 13, in contrast to a low methylation status of intron 13 in the *lactase persistence* haplotype resulting in high lactase expression [221].

We hypothesize that milk-derived miRNAs targeting DNMTs may maintain the low methylation status of intron 13, thus promoting lactase production during the period of breastfeeding. After weaning, the physiological disappearance of milk miRNA-mediated DNMT suppression might downregulate intestinal lactase expression, which is no more required after the lactation period. However, persistent milk consumption in Neolithic humans with a need to handle persisting lactose exposure may have increased natural selection pressure favoring the mutational cytosine > thymidine exchange at intron 13 (-13910C>T), which may contribute to reduced intron 13 cytosine methylation, thereby maintaining persistent high lactase expression.

## 7. Appetite Control and Feeding Reward

**Figure 3:**
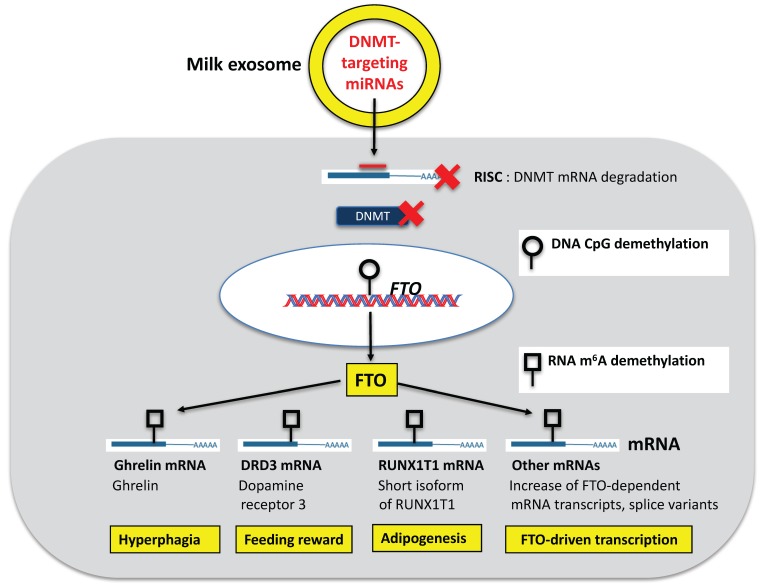
Working model of milk-mediated epigenetic actication of fat mass- and obesity-associated protein (FTO) expression modifying the epitranscriptome. Milk-derived DNMT-targeting miRNAs reduce methylation critical DNA CpG islets thereby increasing FTO gene expression. The RNA m^6^A demethylase FTO erases m^6^A marks on mRNAs, thereby enhancing FTO-dependent mRNA transcription and mRNA splice variant production such as adipogenic short form of RNX1T1. The mRNAs of ghrelin and dopamine receptor 3 (DRD3) are targets of FTO-mediated upregulation. Resulting hyperphagia and feeding rewards support milk intake for infant growth requirements. Via epigenetic upregulation of FTO expression milk regulates the m^6^A-controlled epitranscriptome.

The newborn infant needs continuous access to calories and milk-derived signal transduction for appropriate postnatal growth, which requires active suckling, a strenuous effort for the newborn infant. Epigenetic mechanisms of milk may thus regulate the magnitude of appetite and reward signals in order to guarantee adequate and continuous calorie intake during the postnatal growth phase. Increased expression of the RNA m^6^A demethylase FTO in mice with two additional copies of *FTO* (*FTO-4* mice) exhibited increased hyperphagia and adiposity [227]. Importantly, FTO overexpression reduced ghrelin mRNA m^6^A methylation, concomitantly increasing ghrelin mRNA and protein levels [228] (Figure 3). Ghrelin functions as an orexigenic neuropeptide in the central nervous system and regulates energy homeostasis and reward from food intake [229]. Ghrelin increases appetite by triggering receptors in the arcuate nucleus [230]. The ghrelin receptor is expressed in the brain to regulate feeding, including hypothalamic nuclei involved in energy balance regulation and reward-linked areas such as the ventral tegmental area [231]. Ghrelin signaling at the level of the mesolimbic system is one of the key molecular substrates that provides a physiological signal connecting gut and reward pathways [231]. Ghrelin interacts with neuropeptide Y Y1 and opioid receptors to increase food reward [232]. Milk intake via epigenetic upregulation of FTO may thus trigger ghrelin signaling thereby enhancing appetite and suckling reward to secure postnatal food intake required for postnatal growth and development. The key mechanisms enhancing ghrelin expression of the milk recipient may be increased epigenetic upregulation of hypothalamic FTO expression via milk miRNA-mediated demethylation of the FTO gene. In the offspring of obese female Sprague Dawley rats at weaning, hypothalamic FTO mRNA expression was increased significantly and FTO was correlated with both visceral and epididymal fat mass and hyperphagia [233]. Thus, human milk via FTO-mediated m^6^A-demethylation of ghrelin mRNA may maintain specific orexigenic and reward signals, whilst ensuring appropriate appetite regulation, a developmental requirement adjusting physiological growth trajectories of the human infant (Figure 3).

Persistent uptake of bovine milk exosomal miRNAs may epigenetically enhance long-term orexigenic signaling promoting overgrowth and obesity of the human consumer of cow’s milk. In fact, epidemiological studies confirmed enhanced BMI and linear growth in relation to cow’s milk consumption in children and adolescents [173,234].

Activation of dopaminergic signaling plays an important role in the midbrain and frontal cortex during postnatal development [235,236]. Midbrain dopaminergic neurons in the substantia nigra pars compacta and ventral tegmental area regulate extrapyramidal movement and important cognitive functions, including motivation, habit learning, and reward associations [237]. Variations in *FTO* are the strongest common genetic determinants of adiposity, and may partly act by influencing dopaminergic signaling in the brain leading to altered reward processing that promotes appropriate food intake [238]. Remarkably, *FTO* regulates the activity of the dopaminergic midbrain circuitry [239]. Inactivation of *FTO* impairs dopamine receptor type 2 (D2R) and type 3 (D3R)-dependent control of neuronal activity and behavioral responses [239]. Analysis of global m^6^A modification of mRNAs in the midbrain and striatum of FTO-deficient mice revealed increased m^6^A levels in a subset of mRNAs important for neuronal signaling, including many in the dopaminergic signaling pathway (Table 3) [239].

Conversely, milk-mediated upregulation of FTO stimulated via DNMT-targeting exosomal miRNAs may increase FTO expression and m^6^A demethylation of specific mRNAs promoting dopaminergic transmission. Milk exosome-derived miRNAs may adjust the right epigenetic magnitude of FTO-mediated dopaminergic signaling during the lactation period to maintain an appropriate neuronal growth, self-regulated food intake and feeding reward. Persistently overactivated FTO expression by continued cow’s milk consumption may maintain a state of hyperphagia promoting obesity.

## 8. Intestinal Growth

There is compelling evidence that IECs are able to take up bovine milk exosomes [75,76]. Porcine milk exosomes fed to mice transferred miRNAs, which increased in IECs and modified specific target gene expression promoting IEC proliferation [77]. Lipids are of particular importance for the maintenance and synthesis of cell membranes, which are required for intestinal growth. A key transcription factor of lipid synthesis is SREBP1, which is upregulated via activated AKT-mediated signal transduction. Notably, it has been demonstrated in bovine MECs that the expression of both AKT and SREBP1 underlie epigenetic miRNA-29/DNMT-mediated demethylation of their corresponding promoter regions [122]. Human milk contains and transfers milk stem cells with multilineage differentiation potential to the newborn infant [54,55]. Notably, DNMT inhibition promoted the differentiation of human induced pluripotent stem cells into functional enterocytes [240].

It is thus conceivable that milk-derived DNMT-targeting miRNAs support IEC maturation as well as milk stem cell differentiation into enterocytes, potential contributions for appropriate growth, maturation and function of the infant’s gut.

## 9. Adipogenesis

In humans, new adipocyte formation occurs throughout childhood and adolescence, with fat cell numbers plateauing around the age of 20 years [241]. Approximately 10% of fat cells are renewed annually at all adult ages [241]. Three new mesenchymal phenotypes were expressed in cultures of Swiss 3T3 and C3H/10T1/2CL8 mouse cells treated with the DNMT inhibitor 5-azacytidine. These phenotypes were characterized as contractile striated muscle cells, biochemically differentiated adipocytes and chondrocytes capable of the biosynthesis of cartilage-specific proteins [242]. Mesenchymal stem cells (MSCs) from different tissues may be marked by lineage-specific promoter hypomethylation [243]. Hypomethylation of adipogenic loci in undifferentiated cells may reflect a commitment of these cells to a specific lineage [243]. Uncultured adipose stem cells (ASCs) display hypomethylated promoters of the adipogenic genes leptin (*LEP*), PPAR-γ2 (*PPARG2*), fatty acid-binding protein 4 (*FABP4*), and lipoprotein lipase (*LPL*) [243]. Londoño Gentile et al. [244] recently reported that silencing of DNMT1 can accelerate adipocyte differentiation. DNMT1 gene expression is induced early in 3T3-L1 adipocyte mitotic clonal expansion and is critical for maintenance of DNA and histone H3K9 methylation patterns during this period. However, absence of DNMT1 results in accelerated adipocyte differentiation associated with precocious adipocyte-specific gene expression and lipid accumulation [236]. Later in differentiation, DNMT1 levels decline in an ATP-citrate lyase (ACL)-dependent manner. ACL-mediated suppression of DNMT1 occurs at least in part by promoting expression of miRNA-148a, which represses DNMT1 [244].

miRNA-21, another abundant exosomal miRNA of human, bovine and porcine milk, has recently been shown to enhance adipogenic differentiation from porcine bone marrow-derived mesenchymal stem cells [245]. Lim et al. [246] identified 31 genes whose promoters were significantly differentially methylated between white (WAT) and brown adipogenesis (BAT) at all three time points of differentiation. Among them, five genes belong to the Hox family; their expression levels were anti-correlated with promoter methylation, suggesting a regulatory role of DNA methylation in transcription. Blocking DNA methylation with 5-aza-cytidine increased the expression of these genes, with the most prominent effect on Hoxc10, a repressor of BAT marker expression [246].

Caveolin 1 (Cav-1) is an essential constituent of adipocyte caveolae which binds the β-subunit of the IR and is implicated in the regulation of insulin signaling. During adipocyte differentiation of 3T3-L1 cells the promoter, exon 1 and first intron of the Cav-1 gene undergo a demethylation process that is accompanied by a strong induction of Cav-1 expression, indicating that epigenetic mechanisms must have a pivotal role in this differentiation process [179].

Recent evidence links FTO overexpression to enhanced expression of the pro-adipogenic *short isoform* of the transcription factor RUNX1T1 [152]. FTO controls mRNA splicing by regulating the ability of the splicing factor SRSF2 to bind to mRNA in an m^6^A-dependent manner [152]. The pro-adipogenic short isoform of RUNX1T1 stimulates mitotic clonal expansion of MEFs and thus enhances adipocyte numbers [247]. Overexpression of porcine FTO in differentiating porcine intramuscular preadipocytes was located in the nucleus significantly increased the mRNA levels of adipocyte differentiation transcription factors PPARγ, CCAAT/enhancer binding protein-α (CEBPα), lipoprotein lipase and fatty acid synthase [248]. In human skeletal muscle, FTO mRNA expression positively associates with glucose oxidation rates as well as expression of genes involved in oxidative phosphorylation including PPAR-γ coactivator 1-α (PGC1α) [249] (Table 3). Although genes involved in methylation were differentially regulated in skeletal muscle of *FTO-4* mice, no effect of FTO overexpression on m^6^A methylation of total mRNA was detected [227]. However, an m^6^A hypomethylation state was associated with increased FTO expression in mice fed with high-fat diet, whereas the supplementation of the methyl donor betaine prevented these changes [250].

Milk miRNA-mediated DNMT suppression may thus activate FTO-mediated activation of adipogenic transcription factors such as RUNX1T1, PPARγ, CEBPα, SREBP1, and PGC1α. Milk-mediated exosomal transfer of DNMT-targeting miRNAs might exert inhibitory posttranscriptional activity on adipose tissue DNMT1 expression promoting adipocyte differentiation and adipogenesis.

## 10. Myogenesis

Muscle mass acquisition in the adult human is primarily dependent on mechanical stimuli and active muscle contraction, which activates mTORC1 signaling [251,252]. Repetitive active muscle contractions significantly upregulate the metabolic transcription factor NR4A3 [253]. The newborn infant, however, with a still undeveloped neuromuscular system may depend on other stimuli for muscle cell differentiation and growth. In fact, NR4A3 expression is involved in postnatal development and its expression critically depends on nutritional status [254]. Milk-derived exosomal miRNAs apparently provide the required epigenetic signals for muscle cell differentiation and appropriate muscle protein acquisition. It should be kept in mind that NR4A3 expression is epigenetically induced by NR4A3 promoter demethylation [207]. Genome demethylation with either 5-azacytidine treatment or overexpression of the antisense RNA against DNMT1 induced transdifferentiation of mouse fibroblasts into myoblasts [242,255]. Furthermore, it has been shown that demethylation of the distal enhancer of the MyoD gene and the MyoG promoter is essential for the initiation of the myogenic differentiation program [256,257]. Milk-miRNA-mediated suppression of DNMT1 expression may thus augment myogenesis via epigenetic activation of myogenic transcription factors, which closely interact with mTORC1 signaling [258,259].

miRNA-148a is not only involved in adipogenesis but also enhances myogenic differentiation. Overexpression of miRNA-148a significantly promoted myogenic differentiation of both C2C12 myoblast and primary muscle cells. Blocking the function of miRNA-148a with a 2′-O-methylated antisense oligonucleotide inhibitor repressed C2C12 myoblast differentiation [260]. Rho-associated coiled-coil containing protein kinase 1 (ROCK1), a known inhibitor of myogenesis, has been identified as a direct target of miRNA-148a (Table 2) [261].

Members of the Polycomb group proteins (PcGs), YY1 and Ezh2 together regulate a number of muscle loci including both muscle structure genes and muscle relevant miRNAs [261]. Notably, Rybp, an YY1 interacting PcG protein, is able to target Rybp directly through a conserved binding site on its 3′-UTR. miRNA-29 promotes myogenesis via inhibition of Rybp, which acts as a negative regulator of skeletal myogenesis [262]. It is thus conceivable that milk-derived exosomal miRNA-148a and miRNA-29 support the epigenetic program of myogenesis.

## 11. Osteogenesis

Recent evidence indicates that NRF2 represents a key pathway in regulating bone metabolism. NRF2 expression, which is upregulated by DNMT inhibition [162], is required for normal postnatal bone acquisition in mice [263]. Mice lacking NRF2 exhibited a marked deficit in postnatal bone acquisition, which was most severe at 3 weeks of age when osteoblast numbers were 12-fold less than observed in control animals [263]. Thus, milk exosomal miRNA-mediated suppression of DNMTs may promote NRF2-driven postnatal osteogenesis. HDAC4 and TGFβ3 have been demonstrated to function as negative regulators in chondrocytes and osteoblasts. Importantly, miRNA-29b promotes osteogenesis by directly down-regulating these negative regulators of osteoblast differentiation through binding to target 3′-UTR sequences [264]. Thus, miRNA-29b is a key regulator of development of the osteoblast phenotype by targeting anti-osteogenic factors [264].

RUNX2 is another important regulator of osteogenesis. HDAC4 and HDAC5 deacetylate RUNX2, allowing the protein to undergo Smurf-mediated degradation. Inhibition of HDAC increases RUNX2 acetylation, and potentiates bone morphogenetic protein 2 (BMP-2)-stimulated osteoblast differentiation thereby increasing bone formation [265]. TGFβ inhibits osteoblast differentiation through inhibition of the function of RUNX2 by SMAD3 [266]. HDAC4 or 5 is required for efficient TGFβ-mediated inhibition of RUNX2 function [266]. Notably, bovine milk-derived miRNA-29b, which shares the identical seed sequence as human miRNA-29b, has been shown to increase dose-dependently in the serum of healthy human adults after consumption of pasteurized cow’s milk and increased RUNX2 expression in PBMCs of the milk consumers [79]. It is thus conceivable that milk exosome-derived miRNAs control the epigenetic status of RUNX2 and NRF2 promoting osteogenesis.

Recently, miRNA-21, which is also an abundant miRNA of bovine and human milk, has been shown to promote osteogenic differentiation of human bone marrow-derived stem cells [267]. Elevated expression of miRNA-21 has been demonstrated with an increased differentiation potential in human mesenchymal stem cells (hMSCs) during adipogenesis and osteogenesis [245,268,269]. Thus, milk-derived exosomal miRNAs may have an impact on hMSC differentiation. One of the five most abundant exosomal miRNAs isolated from bone marrow derived mesenchymal stem cells, which is involved in osteogenic differentiation, is miRNA-148a [270].

Taken together, miRNA-148a, the most abundant miRNA of milk, is epigenetically involved in the differentiation of Tregs, adipogenesis, myogenesis and osteogenesis.

## 12. Epidermal Differentiation

Intriguingly, not only bone formation but also epidermal barrier integrity of the skin depends on NRF2 signaling. Late cornified envelope 1 genes are transcriptional targets of NRF2 [271]. There is evidence that already *in utero* amniotic fluid activates NRF2 to improve epidermal barrier function [272]. After birth, milk-derived miRNAs targeting DNMTs may support epigenetic NRF2-mediated expression of cornified envelope proteins crucial for skin barrier function. The epidermal growth factor receptor (EGFR) plays a key role for the regulation of epidermal proliferation [273]. EGFR signaling and protein half-life are tightly regulated. Mitogen-inducible gene 6 (MIG6) is a multiadaptor protein involved in the regulation of receptor tyrosine kinase signaling. MIG6 regulates EGFR signaling and turnover by binding EGFR and directly inhibiting tyrosine kinase activity, increasing clathrin-dependent EGFR endocytosis and trafficking into the lysosome, promoting EGFR degradation [274,275,276]. MIG6 has been identified as a negative regulator of EGFR-mediated skin morphogenesis [277]. Deletion of the mouse gene encoding MIG6 causes hyperactivation of endogenous EGFR and sustained signaling through the MAPK pathway [277]. It is important to mention that MIG6 has been identified as a direct target of miRNA-148a (Table 2) [278]. In this regard, milk-derived exosomal miRNA-148a may promote epidermal proliferation as well as proliferation of other EGFR-dependent cells (Table 4) [279].

## 13. Milk-Mediated Epigenetic Signaling and Diseases of Civilization

There is recent interest in the role of miRNA signaling during the perinatal period for life-long epigenetic programming [18,280]. The great majority of clinical and epidemiological studies demonstrated that cow’s milk consumption during pregnancy increased fetal growth and birth weight of the newborn infant [280].

High birth weight and accelerated postnatal weight gain are associated with an increased risk of obesity. Accelerated infant and childhood weight gain are associated with increased energy intake and diminished satiety response at the age of 5 years [281]. Perinatal programming of energy intake and eating behavior provide a potential mechanism linking early life influences with later obesity, T2DM and cardiovascular disease [281]. Notably, single nucleotide polymorphisms of *FTO*, which are associated with increased FTO expression increase the risk for obesity and T2DM [282,283,284,285,286,287]. FTO expression is not only controlled by the nucleotide sequence but also by epigenetic regulation of *FTO*. Continued uptake of milk-derived exosomes that carry DNMT-targeting miRNAs may represent an overlooked mechanism that modifies early programming of the human epigenome promoting FTO-driven food intake and the development of diseases of civilization such as diabesity, allergy, neurodegenerative diseases, and cancer [135].

Continued consumption of cow’s milk during childhood and adolescence accelerates growth trajectories associated with increased BMI [234], linear growth [173] and early onset of menarche [288]. Accelerated growth during infancy and increased BMI in early life are known to enhance the risk for obesity [289,290,291], T2DM [292,293], allergy [294,295,296] and cancer later in life [297,298,299].

### 13.1. Obesity

Milk-derived miRNA-148a and miRNA-21 are critically involved in adipogenesis. Persistent intake of both adipogenic miRNAs apparently promotes obesity. Remember that miRNA-148a directly targets the pivotal genes regulating triglyceride synthesis (*FAS*), cholesterol homeostasis (*LDLR*), cholesterol efflux (*ABCA1*), and β-oxidation (*CTPA1*) [106]. miRNA-148a via targeting DNMT1 and subsequent promoter hypomethylation enhances adipogenic gene expression including insulin (*INS*), insulin-like growth factor-1 (*IGF1*), caveolin-1 (*CAV1*), leptin (*LEP*), PPAR-γ2 (*PPARG2*), fatty acid-binding protein 4 (*FABP4*), and lipoprotein lipase (*LPL*) [170,174,175,179,243]. Additionally, milk miRNA-148a-mediated FTO promoter demethylation may further enhance RNA transcription of the key adipogenic transcription factors RUNX1T1, PPARγ, CEBPα, and PGC1α via erasing m^6^A marks on their target mRNAs.

Milk-mediated exosomal transfer of DNMT-targeting miRNAs may thus exert inhibitory posttranscriptional activity on adipose tissue DNMT1 expression promoting adipocyte differentiation and adipogenesis. Increased hypothalamic FTO expression in the rat correlated with both visceral and epididymal fat mass and hyperphagia [233]. FTO overexpression in mice led to a dose-dependent increase in body and fat mass, and increased food intake resulting in obesity [147]. FTO overexpression enhanced the expression of the pro-adipogenic *short isoform* of the transcription factor RUNX1T1 [152], which stimulates mitotic clonal expansion of MEFs and thus enhances adipocyte numbers [247]. In addition, overexpression of FTO in porcine intramuscular preadipocytes increased the mRNA levels of adipocyte differentiation transcription factors PPARγ, CCAAT/enhancer binding protein-α (CEBPα), lipoprotein lipase (LPL) and fatty acid synthase (FAS) [248]. Milk-mediated epigenetic overexpression of FTO may thus promote the expression of key adipogenic transcription factors as well as clonal expansion of adipocyte numbers. Persistent milk signaling is thus a critical FTO-related epigenetic mechanism inducing obesity [135].

*FABP4* is another key obesity-associated gene [300]. FABP4, a member of the intracellular lipid-binding protein family, is predominantly expressed in adipose tissue, and plays an important role in maintaining glucose and lipid homeostasis [301]. FABP4 functions as a cytosolic lipid chaperone in macrophages and is involved in regulating macrophage ER stress [302]. Increased expression of FABP4 in plasma and PBMCs has been associated with obesity and atherogenic dyslipidemia [301,303,304]. Furthermore, palmitate activation via FABP4 triggers hepatocellular apoptosis via altered phospholipid composition and steatosis by acylation into complex lipids [305]. Furthermore, FABP4 overexpression has been associated with upregulated expression of pro-inflammatory cytokines including interleukin 6 (IL-6) [306,307]. Indeed, a positive dose-dependent association has been detected between milk intake and increased serum IL-6 levels [308]. Thus, overexpression of FABP4 is involved in critical metabolic and pro-inflammatory aberrations associated with the complex pathogenesis of obesity and steatosis. Importantly, it has recently been demonstrated that miRNA-148a via targeting DNMT1 increased FABP4 promoter hyomethylation thereby enhancing FABP4 expression [243,307]. Thus, we predict that in analogy to FTO the expression of FABP4 may as well underly milk-mediated epigenetic upregulation.

There is recent interest in the role of miRNA-21 in the pathogenesis of obesity and diabetes [309]. Cow’s milk is a rich source of exosomal miRNA-21 [37,48]. It has been demonstrated that miRNA-21 acts as a bidirectional switch in the formation of insulin-producing cells by regulating the expression of target and downstream genes (SOX6, RPBJ and HES1) [310]. Deletion of murine miRNA-21 specifically in hepatocytes indicated a crucial role for hepatic miRNA-21 in metabolic disorders associated with diet-induced obesity. Deletion of miRNA-21 in hepatocytes increased insulin sensitivity and modulated the expression of multiple key metabolic transcription factors involved in fatty acid uptake, de novo-lipogenesis, gluconeogenesis and glucose output [311]. Furthermore, long-term inhibition of miRNA-21 reduced body weight and adipocyte size in db/db mice [312]. Thus, persistent uptake of exosomal miRNA-21 via persistent cow’s milk consumption may enhance the risk for obesity and T2DM [6,135,136].

Taken together, cow’s milk transfers obesogenic and orexigenic miRNAs, predominantly miRNA-148a and miRNA-21, that maintain an epigenetic status that is intimately involved in the pathogenesis of diabesity.

### 13.2. Type 2 Diabetes Mellitus

Early onset of menarche, which is related to milk consumption in early childhood [288], is associated with increased risk of T2DM (extensively reviewed recently [313]). Intake of cow’s milk in contrast to fermented milk products has been associated with incident T2DM as reported in the *EPIC-InterAct Study* including 315,428 European participants and 22,085 cases of T2DM [314]. In contrast to equivalent amounts of meat protein, high intakes of milk increased serum insulin and insulin resistance in 8-year-old boys [315]. The *Physicians’ Health Study* (*n* = 21,660) reported a significant increase of diabetes prevalence from 1.7% to 2.6% in men who consumed ≤1 whole milk serving per week in comparison to participants who consumed ≥2 whole milk serving per week, respectively [316]. Translational evidence linked milk-derived miRNAs to the pathogenesis of T2DM [135,317].

It has been demonstrated that cow’s milk consumption increases miRNA-29b levels in PBMCs in a dose-dependent manner [79]. The miRNA-29 family is among the most abundantly expressed miRNAs in pancreas and liver and is regarded as a diabetogenic risk marker [318,319,320]. Kurtz et al. [321] showed that miRNA-29 was upregulated in the livers of DIO mice and in Zucker diabetic fatty (fa/fa) rats. In this model, miRNA-29 functioned through regulation of the transcription factor FOXA2 (FOXA2-mediated regulation of PPARGC1A, HMGCS2 and ABHD5). Obesity in pregnant sheep leads to increased miRNA-29 expression in the liver tissue of offspring lambs, along with decreased markers of insulin signaling, suggesting fetal programming of miRNA-29 expression [322]. Dooley et al. [171] recently investigated the function of miRNA-29 in glucose regulation using miRNA-29a/b-1 (miRNA-29a)-deficient mice and newly generated miRNA 29b-2/c (miRNA-29c) deficient mice. miRNA-29a was identified as a positive regulator of insulin secretion in vivo, with dysregulation of the exocytotic machinery sensitizing β-cells to overt diabetes after unfolded protein stress. By contrast, in the liver both miRNA-29a and miRNA-29c were important negative regulators of insulin signaling via PI3K regulation. Global or hepatic insufficiency of miRNA-29 potently inhibited obesity and prevented the onset of diet induced insulin resistance [171]. These results confirmed strong regulatory functions for the miRNA-29 family in diabesity. Persistent transfer of milk exosomal miRNA-29 via cow’s milk consumption may thus represent a critical epigenetic factor in the pathogenesis T2DM.

Hypomethylation of specific CpG sites of *FTO* have been reported to enhance FTO expression [154]. In fact, decreased FTO methylation has been demonstrated in pancreatic islets of T2DM patients compared to non-diabetic controls [155]. Furthermore, CpG sites in the first intron of FTO of peripheral blood leukocytes exhibited significant hypomethylation in T2DM patients relative to controls [156].

miRNA-29 via targeting DNMT3B and miRNA-148a via targeting DNMT1 may decrease FTO promoter methylation associated with higher FTO expression resulting in decreased m^6^A levels in mRNAs. In fact, the m^6^A contents in the RNA from T2DM patients and diabetic rats were significantly lower compared with the control groups [323]. Notably, FTO mRNA levels were significantly higher in T2DM patients than in controls and inversely correlated with m^6^A content of mRNAs [323]. Thus, not only SNPs result in FTO overexpression but more frequent epigenetic modifications such as milk-mediated FTO overexpression should be appreciated as key drivers of diabesity.

Increased FABP4 expression has been linked to insulin resistance and T2DM [301,324,325,326]. Expression of FABP4 in the muscle and adipose tissues of T2DM rats were positively correlated [324]. Elevation of circulating FABP4 levels is associated with diabesity, hypertension, cardiac dysfunction, atherosclerosis, and cardiovascular events [325,326]. It is noteworthy to mention that SNPs associated with increased expression of FTO and FABP4 are related to increased milk yield, milk fat and protein content of dairy cows [327,328].

### 13.3. Cancer

Milk consumption has been associated with dose-dependent mortality increase [308]. In contrast, low consumption of milk and other dairy products due to lactose intolerance was associated with decreased risks of lung, breast, and ovarian cancers in a population-based study in Sweden (*n* = 22,788) [329]. A prospective study of 25,892 Norwegian women reported that consumers of 0.75 L or more of full-fat milk daily had a relative risk of 2.91 for breast cancer compared with those who consumed 0.15 L or less [330]. Recent evidence underlines that FTO expression may have a critical role for the risk of breast cancer, especially in HER2-overexpressed breast cancer [331]. Notably, exogenous FABP4 was shown to increase breast cancer cell proliferation [332].

There is compelling evidence for the association of whole milk consumption and prostate cancer (PCa), the most common cancer in men of civilized societies [316,317]. A recent meta-analysis considering 11 population-based cohort studies involving 778,929 individuals demonstrated the existence of a linear dose-response relationship between whole milk intake and increase of PCa mortality risk [333]. Intriguingly, recent evidence links miRNA-148a to the promotion of LNCaP prostate cell growth [334]. Importantly, miRNA-148a targets the largest number of known PCa drivers [334]. The addition of cow milk as an exogenous source of miRNA-148a to LNCaP prostate cancer cells in vitro stimulated PCa cell growth producing an average increase in growth rate of over 30% [335]. EGFR signaling promotes survival of prostate tumor-initiating cells and circulating tumor cells that metastasize to bone [336]. Note that EGFR expression in PCa cells is negatively regulated by MIG6 [337], which is a direct target of miRNA-148a [278].

In contrast to PCa, a meta-analysis involving over 900,000 subjects and over 5200 colorectal cancer (CRC) cases supports an inverse association between *nonfermented milk* consumption and risk of CRC in men [338]. It should be noticed that in contrast to fermented milk, nonfermented milk contains higher amounts of bioactive miRNAs including miRNA-148a, the most abundant miRNA of cow’s milk [63]. Downregulation of miRNA-148a expression plays a critical role in CRC carcinogenesis and progression [339,340,341]. Thus, milk-derived miRNA-148a loaded exosomes may substitute miRNA-148a deficiency in colorectal adenoma cells thereby preventing their further progression to CRC.

In the pathogenesis of PCa, miRNA-148a is apparently a critically ‘oncomiR’ such as miRNA-21 [342,343,344,345], which is one of the earliest identified cancer-promoting ‘oncomiRs’, targeting numerous tumor suppressor genes associated with proliferation, apoptosis and invasion [346,347,348]. Moreover, exosomal miRNA-21 has been considered as potential biomarker of cancer [349]. Furthermore, increased expression of circulating miRNA-21 has been reported in patients with breast cancer [350], lung cancer [350], colorectal carcinoma [350], and hepatocellular carcinoma [351,352,353]. Hepatocellular carcinoma has recently been related to increased consumption of cow’s milk [354].

Exosomal milk-derived miRNA-148a and miRNA-21 may thus provide oncogenic signals inducing an epigenetic landscape for tumorigenesis maintained by the consumption of cow’s milk in the majority of cancers except CRC.

### 13.4. Neurodegenerative Diseases

Accumulating evidence points to the important role of dietary epigenetic regulation in the amyloidopathy Alzheimer’s disease (AD) and the synucleinopathy Parkinson’s disease (PD) [355,356,357,358,359]. Notably, AD and PD are both tauopathies. mTORC1 induces abnormally hyperphosphorylated *tau* proteins, which aggregate resulting in compromised microtubule stability [360]. Abnormally hyperphosphorylated tau aggregates form paired helical filaments in neurofibrillary tangles, a key hallmark of AD and other tauopathies [361]. mTORC1 is involved in regulating tau distribution in subcellular organelles and in the initiation of tau secretion from cells to extracellular space [361]. mTORC1 was activated in the AD brains and the activation level of mTOR signaling correlates with cognitive severity of AD patients [362]. As outlined above, FTO plays a pivotal role for mTORC1 activation [159,160] and milk has been identified as a critical activator of mTORC1-dependent translation [135,136].

#### 13.4.1. Alzheimer’s Disease

AD was recently defined as type 3 diabetes mellitus (T3DM) with the combination of apoE4 homozygosity [355,363]. Carriers of the common FTO rs9939609 A allele polymorphism exhibit a reduction in frontal lobe volume of the brain and an impaired verbal fluency performance [364,365]. A population-based study from Sweden found that carriers of the FTO rs9939609 A allele have an increased risk for incident AD [366]. The FTO risk allele rs9939609A is associated with lower nucleus accumbens volumes, suggesting that the higher body weight of risk-allele carriers might be due to changes within reward-related brain structures [366]. Furthermore, an interaction between FTO and APOE was found, with increased risk for dementia for those carrying both FTO AA and APOE ε4 [367]. Increased levels of brain APOE ε4 mRNA have been detected in AD cases compared to controls with the same allele [368]. The APOE ε4 allele is associated with a gene-dose-dependent increase in AD risk and in the severity of amyloid-β (Aβ) pathology [369], whereas the APOE ε3 allele is thought to protect against Aβ neurotoxicity [369]. A transgenic mouse model of AD expressing human APOE isoforms indicated that different APOE alleles might influence clearing soluble Aβ from the brain [370]. Blocking the apoE/Aβ interaction ameliorates Aβ-related pathology in APOE ε2 and ε4 targeted replacement AD model mice [371].

Interestingly, APOE has recently been demonstrated to be differentially methylated in AD [372]. The three common alleles of APOE, ε2, ε3 and ε4, are defined by two SNPs that reside in the coding region of exon 4, which overlaps with a well-defined CpG island (CGI). Both SNPs change not only the protein codon but also the quantity of CpG dinucleotides, primary sites for DNA methylation. Foraker et al. [372] suggested that the presence of an ε4 allele changes the DNA methylation landscape of the *APOE* CGI and that such epigenetic alteration may contribute to AD susceptibility. To explore the relationship between APOE genotype, AD risk, and DNA methylation of the *APOE* CGI, they evaluated the methylation profiles of *post mortem* brain from 15 AD and 10 control subjects. They observed a tissue-specific decrease in DNA methylation with AD and identified two AD-specific differentially methylated regions (DMRs), which were also associated with APOE genotype. One DMR was completely unmethylated in a subpopulation of genomes, possibly due to a subset of brain cells carrying deviated *APOE* methylation profiles. This study suggests that the APOE CGI is differentially methylated in AD brain in a tissue- and APOE-genotype-specific manner, which might contribute to neural cell dysfunction in AD brain [372]. Genetic variation in introns 1 and 2 of the FTO gene may as well contribute to AD risk [373].

It is conceivable that in analogy to *APOE*, SNPs of *FTO* may modify *FTO* methylation. Remarkably, impaired satiation and increased feeding behavior has been reported in the triple-transgenic AD mouse model [374], which may point to an increased FTO expression. Exosomal transfer of DNMT1-targeting miRNA-148a via cow’s milk consumption may decrease *APOE* and *FTO* methylation. *APOE* and *FTO* SNPs may as well modify the magnitude of functionally important CpG methylations.

#### 13.4.2. Parkinson’s Disease

Several studies show an association between milk consumption and the risk of PD [375,376,377,378,379,380,381]. The largest meta-analysis of 304,193 subjects including 1083 PD cases reported a linear dose-response relationship between milk intake and PD risk, which increased by 17% for every 200 g/day increment in milk intake [380]. Recently, milk intake has been associated with *substantia nigra* neuron loss in decedent brains unaffected by PD [381]. The impairment of DNA methylation has been implicated to represent a crucial mechanism of cognitive aging and related neurodegeneration [382]. The expression of α-synuclein (*SNCA*) is an important factor in the pathogenesis of PD [383,384]. Decreased methylation at *SNCA* intron 1 might contribute to deregulation of α-synuclein expression in sporadic PD cases, highlighting the involvement of aberrant epigenetic mechanisms in PD pathology [383,384]. One of the major hallmarks of PD is the occurrence of intracellular protein deposits in the dying neurons, termed *Lewy bodies*, which contain different proteins, including aggregated α-synuclein and its interacting protein synphilin-1 [385,386]. In a yeast model, α-synuclein inhibited phospholipase D, induced lipid droplet accumulation, and affected vesicle trafficking [387]. A number of common genetic variants have been identified, which contribute to cognitive dysfunction in PD, including variants in catechol-O-methyl-transferase, microtubule-associated protein tau, apoE, mutations in glucocerebrosidase and α-synuclein [388,389]. Importantly, α-synuclein sequesters DNMT1 from the nucleus [390]. In fact, a significant decrease in DNA methylation in the frontal cortex of patients with PD and the related disorder *Dementia with Lewy bodies*, have been associated with the retention of DNMT1 in the cytoplasm [390]. The DNMT1-targeting miRNA-148a plays a critical role for the regulation of neurological development in the brain of the zebrafish [391]. As exosomes are able to cross the blood-brain barrier, it is conceivable that bovine milk exosomes reach human brain cells.

Continued consumption of cow’s milk and persistent uptake of bovine exosomal miRNA-148a, which is identical with human miRNA-148a, may represent an epigenetic mechanism suppressing DNMT1, which via *SNCA* demethylation may increase the expression of α-synuclein, a key aggregating protein in PD (Table 5).

## 14. Metformin

There is compelling evidence that all diseases of civilization, i.e., diabesity, neurodegenerative diseases, breast and prostate cancer are related to overexpression of mTORC1 [392,393,394,395,396,397,398]. We have recently presented evidence that metformin functions at multiple regulatory layers as an inhibitor of mTORC1 signaling [399]. In this regard, attenuation of mTORC1 signaling via metformin is just the opposite of milk-induced activation of mTORC1 signaling [136].

Interestingly, Zhong et al. [400] recently reported that metformin induces genome-wide alterations in DNA methylation by modulating the activity of S-adenosylhomocysteine hydrolase (SAHH). The developmentally regulated H19 lncRNA binds to and inhibits SAHH, the only mammalian enzyme capable of hydrolyzing S-adenosylhomocysteine (SAH). SAH is a potent feedback inhibitor of S-adenosylmethionine (SAM)-dependent methyltransferases that methylate diverse cellular components, including DNA, RNA, proteins, lipids and neurotransmitters [401]. Zhou et al. [402] demonstrated that H19 knockdown activates SAHH, leading to increased DNMT3B-mediated methylation of an lncRNA-encoding gene Nctc1 within the Igf2-H19-Nctc1 locus. Genome-wide methylation profiling revealed methylation changes at numerous gene loci consistent with SAHH modulation by H19 [402]. Intriguingly, metformin acts by upregulating miRNA let-7 through AMPK activation, leading to degradation of H19 lncRNA, which normally binds to and inactivates SAHH. Thus, metformin-induced H19 knockdown activates SAHH, enabling DNMT3B to methylate a subset of genes. It should be noted that all three DNMTs (DNMT1, DNMT3A and DNMT3B) are SAM-dependent. In this regards, milk-miRNA-148a and miRNA-29b-mediated DNMT suppression resulting in DNA demethylation features just the opposite epigenetic signaling compared to metformin-induced DNA methylation.

It should be noticed that metformin’s action on mTORC1 signaling and metformin’s epigenetic modifications in DNA methylation may be interconnected. Promoter methylation of NRF2 attenuates the expression of NRF2, which is a key transcription factor promoting *MTOR* expression [163]. In fact, coadministration of metformin with rapamycin effectively inhibited PCa progression in HiMyc mice [403]. Metformin lowers Ser-129 phosphorylated α-synuclein levels via mTOR-dependent protein phosphatase 2A activation [404]. In human, tau transgenic mice, metformin has been shown to act on tau phosphorylation via mTOR/protein phosphatase 2A signaling and may thus be of therapeutic value for the treatment of AD [405].

## 15. Enhancement of Dairy Milk Yield: A Potential Health Hazard

**Figure 4:**
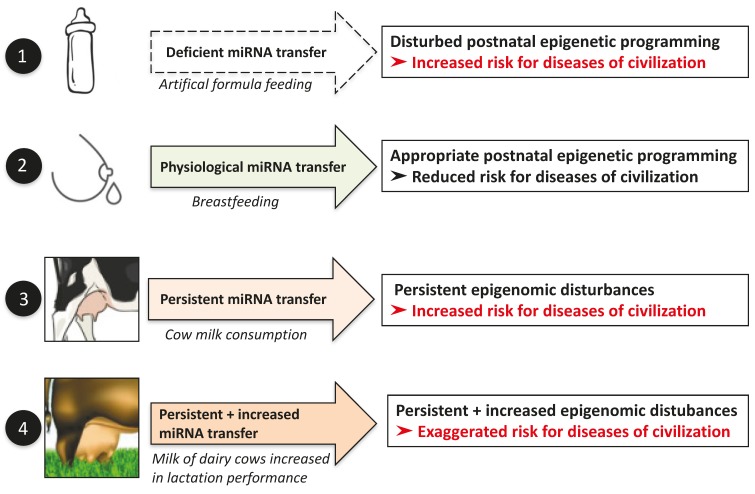
Comparison of milk-miRNA-mediated epigenetic signaling to the human milk recipient. (1) Artifical formula contains only neglectible amounts of bovine miRNAs, which may have an insufficient effect on postnatal epigenetic programming, thus increasing the risk for diseases of civilization; (2) Breastfeeding provides the appropriate epigenetic signaling, which is under control of the human lactation genome, thus reducing the risk for diseases of civilization; (3) Persistent cow milk consumption results in adipogenic, diabetogenic, neurodegenerative, and cancerogenic miRNA signaling; (4) Upregulation of dairy lactation performance increases the burden of milk-derived epigenetic signaling exaggerating the risk of diseases of civilization.

Enhanced milk quality and quantity has become a major selection criterion for the genetic modification of dairy livestock. Epigenetic miRNA-mediated regulations play a major role in bovine mammary gland development and lactation performance [111,122]. Key miRNAs that are abundantly expressed in lactating bovine MECs that promote lactation performance, lipid and protein synthesis include the DNMT-targeting miRNA-148/152- and the miRNA-29 family [116,117,122]. The selection of dairy cows with high expression of these lactogenic miRNAs bears the risk of increased milk exosomal transfer of these DNMT-targeting miRNAs to the human milk consumer [135]. Thus, efforts of dairy research to increase the milk yield of dairy cows may modify the composition and miRNA content of bovine milk exosomes reaching the human milk consumer (Figure 4). Intriguingly, in bovine MEC cultures, the expression of miRNA-148a was stimulated by treatment with dexamethasone, insulin, and prolactin (DIP) [117]. The medium-to-cell expression ratio of miRNA-148a was significantly elevated in these DIP-treated bovine MECs, suggesting extracellular secretion of miRNA-148a into the culture medium after hormonal stimulation of lactation [117]. As already delineated, miRNA-mediated suppression of DNMT expression and consecutive hypomethylation of lactation-related genes (*AKT*, *PPARG*, *SREBP1*, *CSN1S1*, *ELF5*, *GLUT1*) activates lipogenesis, protein synthesis and thus milk yield [108,109,114]. Via exosome transfer, milk of high yield dairy cows may expose the human milk consumer to a most critically overexpressed epigenetic machinery. Maximization of lactation performance over the last decades apparently modified the epigenetic signaling magnitude of cow’s milk further increasing the risk of diseases of civilization (Figure 4). Whereas veterinary medicine unintentionally further increases the burden of miRNA-148a for the human milk consumer, lipidologists are concerned about high expression of miRNA-148a in the context of dyslipidemia and cardiovascular disease and recommend miRNA-148a inhibition as a promising therapeutic approach [406].

## 16. Future Prospects and Conclusions

Milk, mammals’ masterpiece of evolution for maternal-neonatal programming, apparently modifies critical checkpoints of epigenetic regulation of the milk recipient, who under physiological conditions is the newborn infant. Based on our translational insights, we have identified a fundamental epigenetic signaling motive of milk that involves the transfer of DNMT-targeting miRNAs, such as milk’s most abundant miRNA-148a, to the milk recipient. miRNA-mediated DNMT suppression results in hypomethylation and thereby activation of pivotal developmental genes important for metabolic (*INS*, *IGF1*, *CAV1*), immunological (*FOXP3*, *NRA4*), adipogenic (*FTO*, *FABP4*, *CAV1*, *PPARG2*, *SREBP1*, *LPL*), myogenic (*NR4A3*), osteogenic (*NRF2*), and epidermal (*NRF2*) programming. Milk’s epigenetic miRNA signaling predominantly affects stem cell differentiation such as FTO-mediated adipogenesis. In this regard, milk can be regarded as mammals’ natural “doping system” modifying the recipient’s epigenome. In contrast, metformin, the most common biguanide drug for the treatment of T2DM and for the prevention of Western diseases, increases DNA genome-wide DNA methylation. It should be kept in mind that mammalian biology restricts the epigenetic doping of milk to the limited period of lactation and controls this essential system via the action of the well-preserved species-adapted lactation genome.

There is good reason to conclude that milk’s epigenetic machinery during postnatal life is of critical importance for life-long metabolic programming. It is conceivable that human milk’s epigenetic signaling is beneficial for the infant’s development and health. It is thus of critical concern that milk’s miRNA-based epigenetic signaling is deficient or functionally absent in artificial infant formula bearing the risk of inappropriate metabolic and immunological programming.

We conclude that the persistent abuse of milk’s epigenetic signaling via continued consumption of pasteurized cow’s milk increases the risk for diseases of civilization. Persistent hypomethylation of critical genes such as FTO are associated with the pathogenesis of diabesity, neurodegenerative diseases, and common cancers. Whereas fermentation of milk impairs milk’s exosomal miRNAs, pasteurized, refrigerated cow’s milk still pollutes abundant miRNAs with epigenetic potential into the human food chain. In fact, since the 1950s with the widespread distribution of household refrigerators the incidence of non-communicable diseases of civilization increased steadily. A deeper understanding of milk’s epigenetic capabilities makes “non-communicable” diseases to be communicable. In this regard, we are afraid of commercial efforts enforcing lactation performance in dairy cows which we claim to represent a serious health hazard modifying the human epigenome and epitranscriptome. Whereas pasteurization was formerly introduced to remove pathogenic bacteria from milk, we urge to remove milk’s DNMT-targeting miRNAs from the human food chain. The elimination of commercial milk’s epigenetic machinery but its introduction into artificial infant formula will be two promising approaches for the prevention of Western diseases of civilization.

## Figures and Tables

**Table 1:** Evidence for milk exosome miRNA transfer into the systemic circulation.

The majority of milk exosomes is secreted by mammary epithelial cells	[14]
Milk exsomes resist intestinal degradation	[38,40,43,61,62,66]
Milk exosomes are taken up by intestinal epithelial cells	[75,76,77]
Milk exosomes are taken up by vascular endothelial cells	[78]
Increased serum levels of milk-derived miRNAs during lactation	[41,46]
Dose-dependent increase of miRNA-29b and miRNA-200c in the serum of cow’s milk consumers	[79]
Increase of miRNA-29b and miRNA-200c in peripheral blood mononuclear cells of human volunteers 6 h after commercial milk intake	[79]
Increased expression of RUNX2, a regulatory target of miRNA-29b, in PBMCs of healthy humans after cow’s milk consumption	[79]
Detection of bovine milk exosomes in murine splenocytes	[75]
Predicted role of milk miRNAs in organismal development and organ maturation	[17,80,81]

**Table 2:** High complementarity of seed sequences between human and bovine DNMT-targeting miRNAs.

Human miRNAs Targeting DNMTs	Bovine miRNAs Targeting DNMTs
**hsa-miRNA-148a-5p** 6-aaaguucugagacacuccgacu-27 **hsa-miRNA-148a-3p** 44-ucagugcacuacagaacuuugu-65	**bta-miRNA-148a-3p** 44-ucagugcacuacagaacuuugu-65
**hsa-miRNA-21-5p** 8-uagcuuaucagacugauguuga-29 **hsa-miRNA-21-3p** 46-caacaccagucgaugggcugu-66	**bta-miRNA-21-5p** 8-uagcuuaucagacugauguugacu-31 **bta-miRNA-21-3p** 47-aacagcagucgaugggcugucu-68
**hsa-miRNA-29b-1-5p** 10-gcugguuucauauggugguuuaga-33 **hsa-miRNA-29b-1-3p** 51-uagcaccauuugaaaucaguguu-73	**bta-miRNA-29-1-3p** 51-uagcaccauuugaaaucaguguu-73

**Table 3:** Selected mRNAs upregulated via FTO-mediated m^6^A demethylation.

mRNA	Function	References
RUNX1T1	Promotion of adipogenesis, mitotic clonal expansion increasing adipocyte numbers	[152,247]
PPARγ	Promotion of adipogenesis	[248]
CEBPα	Promotion of adipogenesis	[248]
PGC1α	Promotion of adipogenesis	[249]
Ghrelin	Increased ghrelin mRNA and protein expression, increased orexigenic signaling	[228]
Dopamine receptor 2 and 3	Increased dopaminergic signaling potentially involved in feeding reward	[239]

**Table 4:** Selected target mRNAs of miRNA-148a.

Target	Reported Biological Effects of miRNA-148a	References
DNMT1	Reduced maintenance DNA methylation during cell division	[118,244]
DNMT3B	Reduced de novo DNA methylation	[121]
ABCA1	Reduced reverse cholesterol transport, risk of dyslipidemia	[106]
LDLR	Reduced hepatic uptake of LDL, risk of dyslipidemia	[106]
CPT1A	Reduced mitochondrial fatty acid β-oxidation, risk of dyslipidemia	[106]
MIG6	Reduced inhibition of EGFR, increased cell proliferation	[278]
ROCK1	Reduced suppression of myogenesis, enhanced myogenesis	[261]

**Table 5:** Genes with increased DNA CpG demethylation-dependent expression.

Gene	Functions	References
*FTO*	Increased RNA m^6^A demethylation, resulting in increased transcription, generation of adipogenic splice variant (short form) of RUNX1T1	[152,154,155,156]
*INS*	Increased insulin expression, activation of mTORC1, increased glucose uptake, anabolism	[170]
*IGF1*	Increased IGF-1 expression, activation of mTORC1, promotion of growth and GH signaling	[174,175]
*CAV1*	Stimulation of insulin- and IGF-1 receptor signal transduction, promotion of adipocyte differentiation	[179]
*FABP4*	Adipogenic differentiation	[243]
*LPL*	Adipogenic differentiation	[243]
*NRF2*	Increased expression of mTOR, RagD, promotion of mTORC1 signaling, promotion of osteogenesis	[162,163,164,165,166]
*NR4A3*	Promotion of myogenesis and FoxP3 expression	[201,202,207,253]
*FOXP3*	Increased FoxP3 expression, differentiation and stable expression of regulatory T cells, induction of immune tolerance, prevention of allergy	[183,184,185,186,187]
*APOE*	Decreased isotype-specific APOE methylation in brains of patients with Alzheimer’s disease	[372]
*SNCA*	Decreased methylation at *SNCA* intron 1 in patients with Parkinson’s disease	[383,384]

## Abbreviations

AGO2: argonaute 2; AKT: V-AKT murine thymoma viral oncogene homolog; ASC: adipose stem cells; BAT: brown adipose tissue; BCAA: branched-chain amino acid; BMI: body mass index; BMP: bone morphogenetic protein; CDX2: caudal-type homeobox transcription factor 2; CMA: cow’s milk allergy; CRC: colorectal cancer; DMR: differentially methylated region; DNMT: DNA methyltransferase; EGFR: epidermal growth factor receptor; EMT: epithelial-mesenchymal transition; ESCRT: endosomal sorting complex required for transport; EV: extracellular vesicle; FABP4: fatty acid binding protein 4; FAS: fatty acid synthase; FoxP3: forkhead box P3; FTO: fat mass- and obesity-associated gene; GLUT1: glucose transporter 1; HNRNPA2B1: heterogeneous nuclear ribonucleoprotein A2B1; HSP: heat shock protein; HuR: human antigen R; IDAX: inhibitor of DVL/axin complex; IgE: immunoglobulin E; IGF-1: insulin-like growth factor-1; IGF1R: insulin-like growth factor-1 receptor; IκBα: nuclear factor κB inhibitor α; IL: interleukin; ILV: intralumial vesicles (ILVs); INS: insulin gene; LCT: lactase gene; m^6^A: *N*^6^-methyladenosine; LPL: lipoprotein lipase; METTL: methyltransferase-like; MFG: milk fat globule; MFGM: milk fat globule membrane; miRNA: micro-ribonucleic acid; ILV: intralumial vesicles; MIG6: mitogen-inducible gene 6; mTORC1: mechanistic target of rapamycin complex 1; MVB: multi-vesicular body; NEC: necrotizing enterocolitis; NF-κB: nuclear factor κB; p53: transformation-related protein 53; NR4A3: nuclear receptor subfamily 4, group A, member 3; NRF2: nuclear factor erythroid 2-related factor 2; PCa: prostate cancer; PCNA: proliferating cell nuclear antigen; NFKBI: nuclear factor of κ light chain gene enhancer in B cells inhibitor α; PBMC: peripheral blood mononuclear cell; PI3K: phosphatidylinositol 3-kinase; PGC1α: PPAR-γ coactivator 1-α; PPARγ: peroxisome proliferator-activated receptor-γ; PP2A: protein phosphatase 2A; RASGRP1: Ras guanyl nucleotide-releasing protein-1; RNA: ribonucleic acid; RISC: RNA-induced silencing complex; ROCK1: Rho-associated coiled-coil containing protein kinase 1; RUNX1T1: Runt-related transcription factor 1, translocated to, 1; SAH: S-adenosylhomocysteine; SAHH: S-adenosylhomocysteine hydrolase; SAM: S-adenosylmethionine; SCD1: stearoyl-CoA desaturase 1; SNP: single nucleotide polymorphisms; SREBP-1: sterol regulatory element-binding protein 1; TCR: T cell receptor; T2DM: type 2 diabetes mellitus; T3DM: type 3 diabetes mellitus; TET: ten-eleven-translocation; TGFβ: transforming growth factor-β; TNF: tumor necrosis factor; TSDR: Treg-specific demethylated region; UTR: untranslated region; WAT: white adipose tissue; YBX1: RNA-binding protein Y-box protein 1.
